# Supplementary material for: The boundary-spanning mechanisms of Nobel Prize winning papers
Source: PLoS One. 2021 Aug 11;16(8):e0254744. doi: 10.1371/journal.pone.0254744 (PMC8357150; doi:10.1371/journal.pone.0254744)
Supplement: Attachment — Submitted filename: Response to Reviewers.pdf [file pone.0254744.s001.pdf]

Yakub Sebastian[1] and Chaomei Chen[2]
[1] College of Engineering, IT & Environment, Charles Darwin University, Australia
[2] College of Computing & Informatics, Drexel University, PA, USA
Correspondence: yakub.sebastian@cdu.edu.au

Yiming Tang, PhD
Academic Editor
PLOS ONE

May 22, 2021

RE: Response to Reviewers for manuscript PONE-D-21-07994

Dear Dr. Tang

Thank you for considering our manuscript and giving us the opportunity to respond to the reviewer's comments. We also thank the reviewer for providing highly constructive feedback.

In this Response to Reviewers, we describe our responses to the reviewer's open comments on pages 1 – 3 in this letter. It is then followed by responses to minor editorial comments with regards to the dataset availability on page 4.

All changes made to the manuscript are highlighted in the document "Revised Manuscript with Track Changes".

We hope we satisfactorily addressed any of the concerns raised and look forward to having the manuscript accepted into the journal. Thank you for your time.

Sincerely,

Yakub Sebastian and Chaomei Chen

Reviewer's open comments

1. My only major comment is about the framing of its contribution. There have been many citation-based predictors related to Nobel Prize (on a related note, the authors may also want to cite "Network Signatures of Success: Emulating Expert and Crowd Assessment in Science, Art, and Technology"). The current version distinguishes from itself from these works in timing, i.e. "leading indicators of breakthrough that can be computed at the time of a paper's publication". Yet in my opinion, the difference between the two approaches may be even more fundamental – the new analysis presented here is purely based the stock of knowledge, rather than scientific impact or recognition received from future works. Maybe the authors can come up with better words/phrases, but I would recommend thinking a little bit more around the conceptualization.

Our response:
We thank the reviewer for pointing us to the related work Zakhlebin, I. and Horvát, E.Á., 2017, November. Network signatures of success: Emulating expert and crowd assessment in science, art, and technology. In *International Conference on Complex Networks and their Applications* (pp. 437-449). Springer. Correspondingly, we added a review of this work in section "Network properties and structural variations", lines 138 – 146 with a new reference [41] corresponding to it.

As suggested by the reviewer, we further elaborated the conceptualization and impacts of our research contributions on lines 190 – 202 and 696 – 734. We also added new references [46], [47] and [69] in support of the arguments.

2. The readers may not be extremely familiar with SVA, so it might be better to provide more explanations for technical terms. For one, the word "cluster" can be ambiguous in network literature – some use it to indicate connected components while others use it to any communities in community detection tasks (which seem to be the case here).

Our response:
We added two new paragraphs on lines 290 – 306 that provide detailed explanations of two key terminologies underlying SVA: **co-citation network** and **clusters**. As correctly observed by the reviewer, the precise meaning of the term `cluster' in this manuscript carries a similar meaning to `community' in community detection.

We also included a new reference [57], which provides the interested novice readers with extra insights into the theory and intuition behind co-citation networks.

3. I wonder how scalable this approach would be. For example, if we want to run an analysis over all Nobel-prize winning papers, what is the rough estimation of manual efforts one should spend? To be clear I'm not questioning the practical value of this method, but adding some more comments around this would be extremely helpful for future studies (either a highlight of its high scalability or a sentence in limitation should be good).

Our response:
We added 1 paragraph under 'Limitation' section on lines 846 – 857 on the scalability of our approach and current limitations.

4. For discussion of future works, a promising direction would be engaging more advanced network analysis tools (e.g. high-order indexes as well as network embeddings) to better understand the predictability of network-based approaches. Again I'm not asking for additional analyses here, but having some discussions around this should make the paper more relevant for a broader range of audience.

Our response:
We added 1 paragraph under 'Limitation' section on lines 858 – 862, suggesting that recent network analysis paradigms (such as network embedding, heterogeneous network representation, and knowledge graph learning) may provide possible alternatives for measuring boundary-spanning qualities of Nobel Prize winning papers. New references [71 – 73] were added in support of these.

Dataset availability

[3.] Have the authors made all data underlying the findings in their manuscript fully available?

The PLOS Data policy requires authors to make all data underlying the findings described in their manuscript fully available without restriction, with rare exception (please refer to the Data Availability Statement in the manuscript PDF file). The data should be provided as part of the manuscript or its supporting information, or deposited to a public repository. For example, in addition to summary statistics, the data points behind means, medians and variance measures should be available. If there are restrictions on publicly sharing data—e.g. participant privacy or use of data from a third party—those must be specified.

Reviewer #1: No

Our response:
The datasets we generated and analyzed for this study are freely accessible from Figshare at https://figshare.com/s/5e0e279c51f6de9df947 . Further information about the dataset provision and access is provided in the S1 file (Supporting Information).

Acknowledgment

We added a thank you note to the anonymous reviewer.

End of Response to Reviewers
